# Estimating the marginal cost of a life year in Sweden’s public healthcare sector

**DOI:** 10.1007/s10198-019-01039-0

**Published:** 2019-02-22

**Authors:** Jonathan Siverskog, Martin Henriksson

**Affiliations:** Centre for Medical Technology Assessment, Department of Medical and Health Sciences, Linköping University, 581 83 Linköping, Sweden

**Keywords:** Opportunity cost, Threshold, Healthcare expenditure, Mortality, Life expectancy, Cost-effectiveness analysis, C32, C33, C36, I10, I18

## Abstract

Although cost-effectiveness analysis has a long tradition of supporting healthcare decision-making in Sweden, there are no clear criteria for when an intervention is considered too expensive. In particular, the opportunity cost of healthcare resource use in terms of health forgone has not been investigated empirically. In this work, we therefore seek to estimate the marginal cost of a life year in Sweden’s public healthcare sector using time series and panel data at the national and regional levels, respectively. We find that estimation using time series is unfeasible due to reversed causality. However, through panel instrumental variable estimation we are able to derive a marginal cost per life year of about SEK 370,000 (EUR 39,000). Although this estimate is in line with emerging evidence from other healthcare systems, it is associated with uncertainty, primarily due to the inherent difficulties of causal inference using aggregate observational data. The implications of these difficulties and related methodological issues are discussed.

**Electronic supplementary material:**

The online version of this article (10.1007/s10198-019-01039-0) contains supplementary material, which is available to authorized users.

## Introduction

In practise, the decision to reimburse an intervention is often informed by judging its incremental cost-effectiveness ratio (ICER) against a cost-effectiveness threshold. Although imperative for resource allocation decisions and the interpretation of cost-effectiveness analysis, this threshold value has received remarkably little attention up until recently [1]. Sweden is no exception, and despite a long tradition of using cost-effectiveness analysis as an input into healthcare decision-making, the criteria for when an intervention is considered too expensive are vague. It has been argued that a threshold should represent the opportunity cost of healthcare resource use [2] and most commonly this is construed either as private consumption forgone or health forgone. These two conceptions of opportunity cost are often referred to as the demand-side threshold (v-threshold), which tells us the consumption value of health gains, and the supply-side threshold (k-threshold), which indicates the marginal cost at which health could be generated if resources were not re-allocated to fund the evaluated intervention. Whether the demand-side or supply-side threshold is deemed more appropriate depends, among other things, on the objective function and the constraints of the relevant authority. However, regardless of these aspects, there seems to be consensus in the literature that an estimate of the opportunity cost in terms of health forgone is often required [1, 2]. If resources are not readily transferrable between sectors, we cannot know whether reimbursement or approval decisions are expected to increase or decrease population health (by displacing other more productive healthcare services) without such an estimate. Furthermore, as noted by Brouwer et al. [1], even if resources are assumed (at least partly) transferable between sectors, an estimate of the supply-side threshold would be useful for understanding the discrepancy between what we would like to spend and what we are actually spending to gain health. Although estimates are emerging in the literature [3–5], there is still no empirical estimate of the supply-side threshold for Sweden and many other countries where cost-effectiveness analysis is an important aspect of healthcare decision-making. The approach in available studies is to derive the threshold from the marginal effect of healthcare expenditure on mortality. There is, of course, already an abundance of studies on this relationship; see, e.g. Nolte and McKee [6] for a review or Gallet and Doucouliagos [7] for a meta regression analysis. As noted by Gravelle and Backhouse [8], however, it is important for such estimation to take into account that expenditure is unlikely to be exogenous with respect to mortality, which most past studies fail to do. Therefore, the relevant literature on this approach to supply-side thresholds is, so far, quite sparse.

To the best of our knowledge, the study by Lichtenberg [9] is the first to express the relationship between healthcare expenditure and mortality as a cost per life year gained. It uses a time series on life expectancy at birth and public health expenditure in the United States and estimates a geometric lag model to derive a cost per life year of USD 9640. The issue of endogeneity is to some extent addressed by the model lag-structure and Granger causality testing. Martin et al. [10] estimate two disease-specific thresholds, one for cancer care at GBP 13,137 per life year and one for circulatory disease at GBP 7979 per life year. Their study considers a cross-section of years of life lost (YLL) and NHS expenditure for English primary care trusts. The relationship is estimated by two-stage least squares (2SLS), where the proportion of households that are lone pensioner households and the proportion of the population providing unpaid care act as instrumental variables for expenditure. Using data on EQ-5D scores for ICD-10 categories, average health-related quality of life (HRQoL) weights are calculated for cancer and circulatory disease to adjust the estimates to costs per quality-adjusted life year, QALY (GBP 19,070 and GBP 11,960, respectively). This approach is reapplied to ten programmes of care by Martin et al. [11]. Claxton et al. [3] build on this work to estimate an overall threshold for the English NHS at GBP 12,946, by also estimating the budget elasticity of expenditure for all programmes of care. This also attempts to include pure HRQoL effects by considering the QALY burden of disease. Claxton et al. [12] further consider a different set of instruments suggested by Andrews et al. [13] to re-estimate some mortality effects, but do not derive a new threshold.

More recently, Edney et al. [4] estimate the effect of healthcare expenditure on YLL from a cross-section of small geographical areas in Australia. They also use the proportion of the population providing unpaid care as an instrument for expenditure and derive a threshold for the Australian health system at AUD 28,033 per QALY. YLL are adjusted for HRQoL using age- and gender-specific weights. Effects of expenditure on HRQoL are captured using a panel on individual utility scores from the SF-6D. Using a range of demographic and socioeconomic controls, remaining temporal variation is assumed to be due to healthcare expenditure. Vallejo-Torres et al. [5] use a panel on healthcare expenditure and life expectancy for regions in Spain to derive a threshold of EUR 22,000–25,000 per QALY. In their study, the percentage of total expenditure allocated to health care is considered as a potential instrument, but they find that expenditure does not appear to be endogenous. It is argued that large budget cuts during the period of study might have led to exogenous variation in expenditure. Life expectancy is adjusted for HRQoL using age-, gender-, year-, and region-specific HRQoL weights. The weights are estimated from three cross-sections on individual EQ-5D scores during the sample period.

Existing estimates provide a point of reference, but the supply-side thresholds for other healthcare systems are not readily transferrable to Sweden. In this study, we therefore seek to estimate the marginal cost of a life year in Sweden’s public healthcare sector using aggregate level data on healthcare expenditure (henceforth abbreviated HCE in equations) and mortality through a time series approach and a panel data approach, both of which address the issue of endogeneity. We find estimation with a time series approach unfeasible due to reversed causality. However, through panel instrumental variable estimation, we are able to derive a marginal cost per life year of about SEK 370,000 (EUR 39,000). There are no data available on variation in HRQoL, but we carry out a few back-of-the-envelope calculations, suggesting a cost per QALY of about SEK 430,000 (EUR 45,000) or SEK 180,000 (EUR 19,000) when adjusting mortality for quality of life and also accounting for pure quality-of-life effects. Besides providing a first estimate of the opportunity cost of re-allocating healthcare resources in Sweden, we contribute to the existing literature by discussing some methodological issues associated with estimating the relationship between mortality and healthcare expenditure and the choice of instrumental variables. Section “Measures of healthcare expenditure and mortality” measures of healthcare expenditure and mortality. In “Methods”, we describe the methods used to analyse the relationship between these measures, while section “Results” reports the results from our analyses. Section “Discussion and conclusion” discusses some issues related to instrument validity and the use of aggregate data, and their implications for the credibility of our estimate.

## Measures of healthcare expenditure and mortality

Sweden is organised into 21 regional councils (or county councils) responsible for primary and secondary care. Whether provided by the councils themselves or by private actors, this care is financed through regional taxes, central government transfers, and to a very small extent, patient fees.^1^ Notable examples of expenditure falling outside the budgets of the regional councils are care for the elderly and disabled, which is mainly the responsibility of the local municipalities, dental care, largely financed through household out-of-pocket payments, patient co-payments for prescription pharmaceuticals, and over-the-counter medicines. According to the OECD System of Health Accounts (SHA) 1.0 definition of healthcare [15], regional councils accounted for 72% of total healthcare expenditure in 2010. Other sources of funds were households (17%), municipalities (8%), and central government (2%). Private insurance, enterprise, and non-profit financing schemes accounted for only 1% of total expenditure [16]. Following the redefinition of long-term healthcare in SHA 2011, which includes activities of daily living for the elderly and disabled, total healthcare expenditure as share of GDP increased by 1.2% points [17, 18]. According to this definition, regional councils accounted for 57% and municipalities for 25% of total healthcare expenditure in 2011.

For the purpose of our analyses, we construct a time series on national healthcare expenditure and a panel on regional healthcare expenditure. At the national level, we consider current government healthcare expenditure per capita for 1970–2016 from the OECD SHA. This includes all central, regional, and municipal government expenditure on healthcare net of capital investment [17]. Since OECD constant prices are calculated using the GDP deflator, we attempt to adjust for the relative price level of healthcare services. This is done by combining the regional council price index from the Swedish Association of Local Authorities and Regions (SALAR) and the pharmaceutical price index from the Swedish eHealth Agency. At the regional level, we use regional council healthcare expenditure per capita for 20 regions between 2003 and 2016^2^ from the municipality and region database (KOLADA), adjusted by the regional council price index and the regional healthcare wage index. Here, healthcare expenditure is defined as gross expenditure on healthcare net of internal revenue and remuneration for services provided to other regions. In both series, the accounting practice is to include the cost of capital at the point of consumption, i.e. capital consumption or depreciation. Details on the price and wage indices are provided in the supplement (Online Resource 1).

**Fig. 1:**
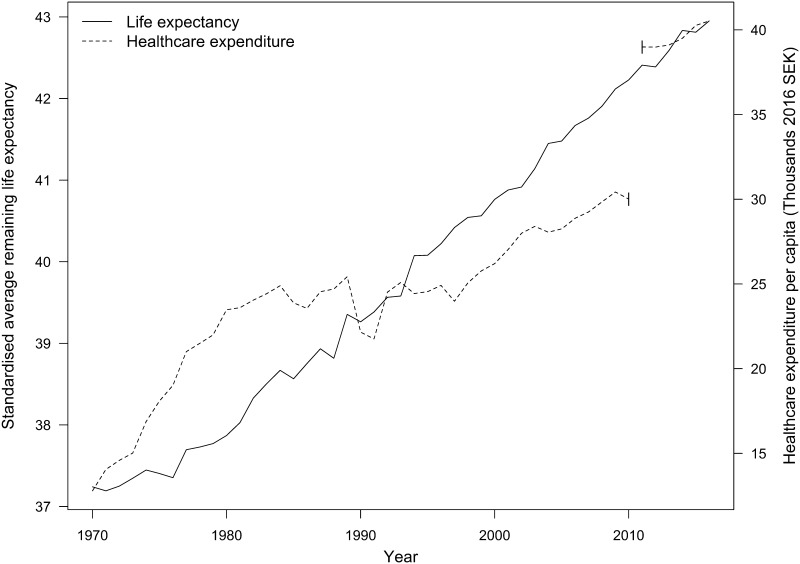
Remaining life expectancy and public healthcare expenditure per capita, 1970–2016

**Fig. 2:**
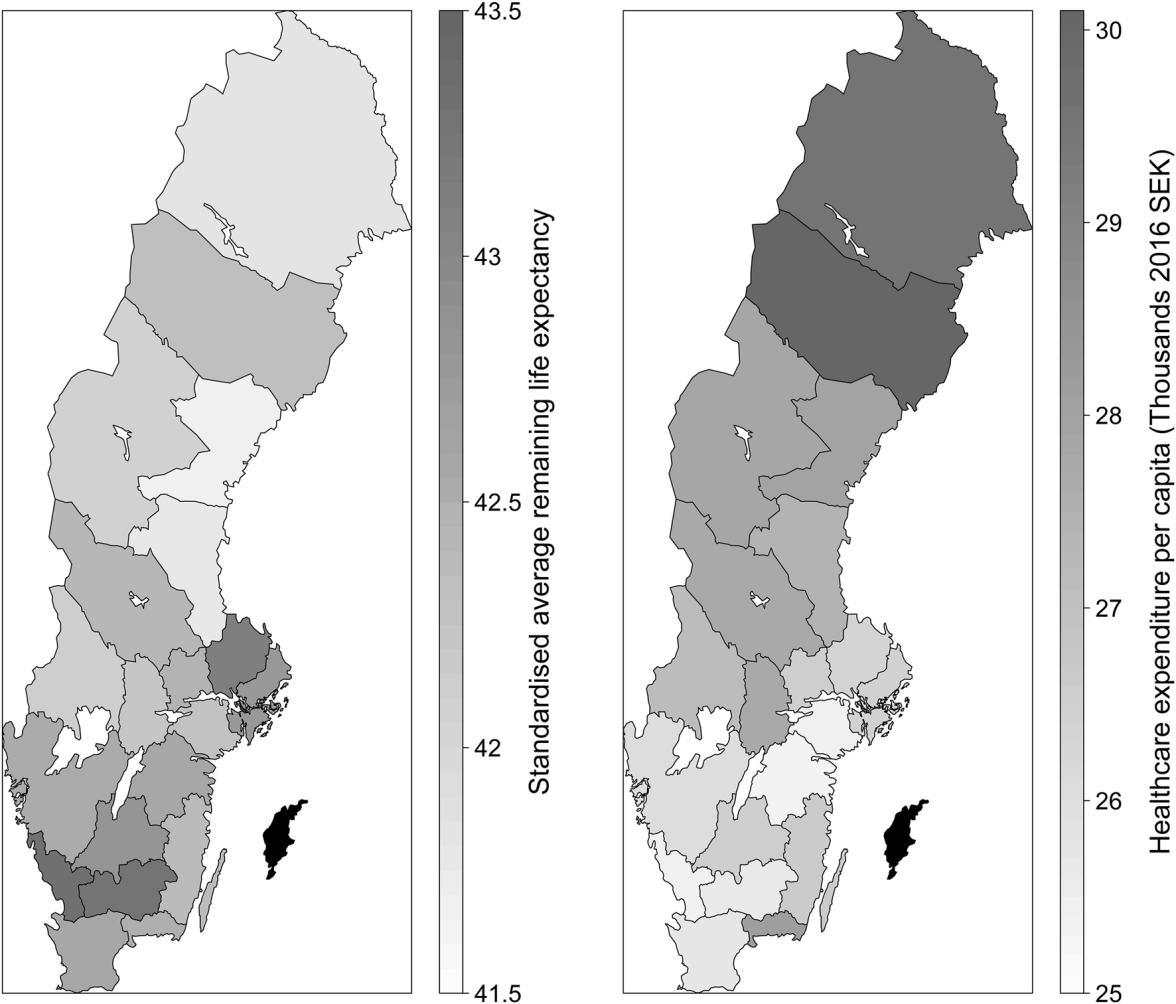
Regional variation in life expectancy and expenditure, 2003–2016 averages

Regarding mortality, previous work has considered either years of life lost [3, 4] or life expectancy [5, 9]. Since the construction of YLL requires estimates of life expectancy, the latter would seem preferable, as it is in fact already an aggregate measure of contemporaneous mortality, despite the misnomer. At the national level, we collect data on remaining life expectancy (RLE) by age and gender for 1970–2016 from Statistics Sweden. At the regional level, we calculate the same measure using the number of deaths by region, year, gender, and age according to birth year; also from Statistics Sweden. First, age- and gender-specific mortality rates are calculated. Then, mortality rates are used to derive remaining life expectancy by age and gender. Finally, to produce a single aggregate measure of mortality, we calculate standardised average remaining life expectancy (ARLE) using national population weights for 2016. In this way, we capture regional and temporal variations in mortality that are not affected by either type of variation in the age and gender structure of the population. The time series on life expectancy and expenditure are plotted in Fig. 1. Regional variation is visualised in Fig. 2. Data construction is detailed in Online Resource 1.

## Methods

### Time series analysis

A common approach to causal inference in time series analysis is to ignore the structural relationship and regard all variables as endogenous within a reduced form vector autoregressive (VAR) framework, where it is possible to test for Granger [19] causality. We estimate a bivariate VAR model, which can be expressed by the following equations:

1 $$\ln {\text{ARL}}{{\text{E}}_t}=\sum\limits_{{p=1}}^{P} {{\phi _p}\ln {\text{ARL}}{{\text{E}}_{t - p}}} +\sum\limits_{{p=1}}^{P} {{\psi _p}\ln {\text{HC}}{{\text{E}}_{t - p}}+\varvec{d}^{\prime}\varvec{\gamma} +{u_t}} ,$$

2 $$\ln {\text{HC}}{{\text{E}}_t}=\sum\limits_{{p=1}}^{P} {{\theta _p}\ln ARL{E_{t - p}}} +\sum\limits_{{p=1}}^{P} {{\pi _p}\ln {\text{HC}}{{\text{E}}_{t - p}}+\varvec{d}^{\prime}\varvec{\lambda} +{v_t}} ,$$

where $P$ is the number of lags, ${\varvec{d}}$ is an optional vector of exogenous variables, and ${u_t}$ and ${v_t}$ are Gaussian white noise error terms. In ***d***, we include a constant and an impulse dummy for 2011 to account for the break in the expenditure series. Toda and Yamamoto [20] show that the appropriate way to test for Granger causality is to determine the optimal lag-length (${P^ \star }$) and estimate a VAR(${P^ \star }+m$) in levels, where $m$ is the maximum order of integration. We determine this using the augmented Dickey–Fuller (ADF) test and Kwiatkowski–Phillips–Schmidt–Shin (KPSS) test. We then impose the restrictions ${\psi _1}={\psi _2}= \cdots ={\psi _{{P^ \star }}}=0$ and ${\theta _1}={\theta _2}= \cdots ={\theta _{{P^ \star }}}=0$, to see if it is possible to reject that the history of one variable explains the future values of the other variable. The results will indicate either unidirectional, bidirectional, or no Granger causality. In the case of Granger causality, it is possible that there is a long-run relationship between the variables. We test for the presence of such a relationship using the Johansen [21] cointegration test. Assuming that causality runs from expenditure to life expectancy, the long-run relationship from the VAR model’s error correction form could be interpreted as the expenditure elasticity of life expectancy. All time series analyses are performed in Eviews 9.

### Panel data analysis

**Fig. 3:**
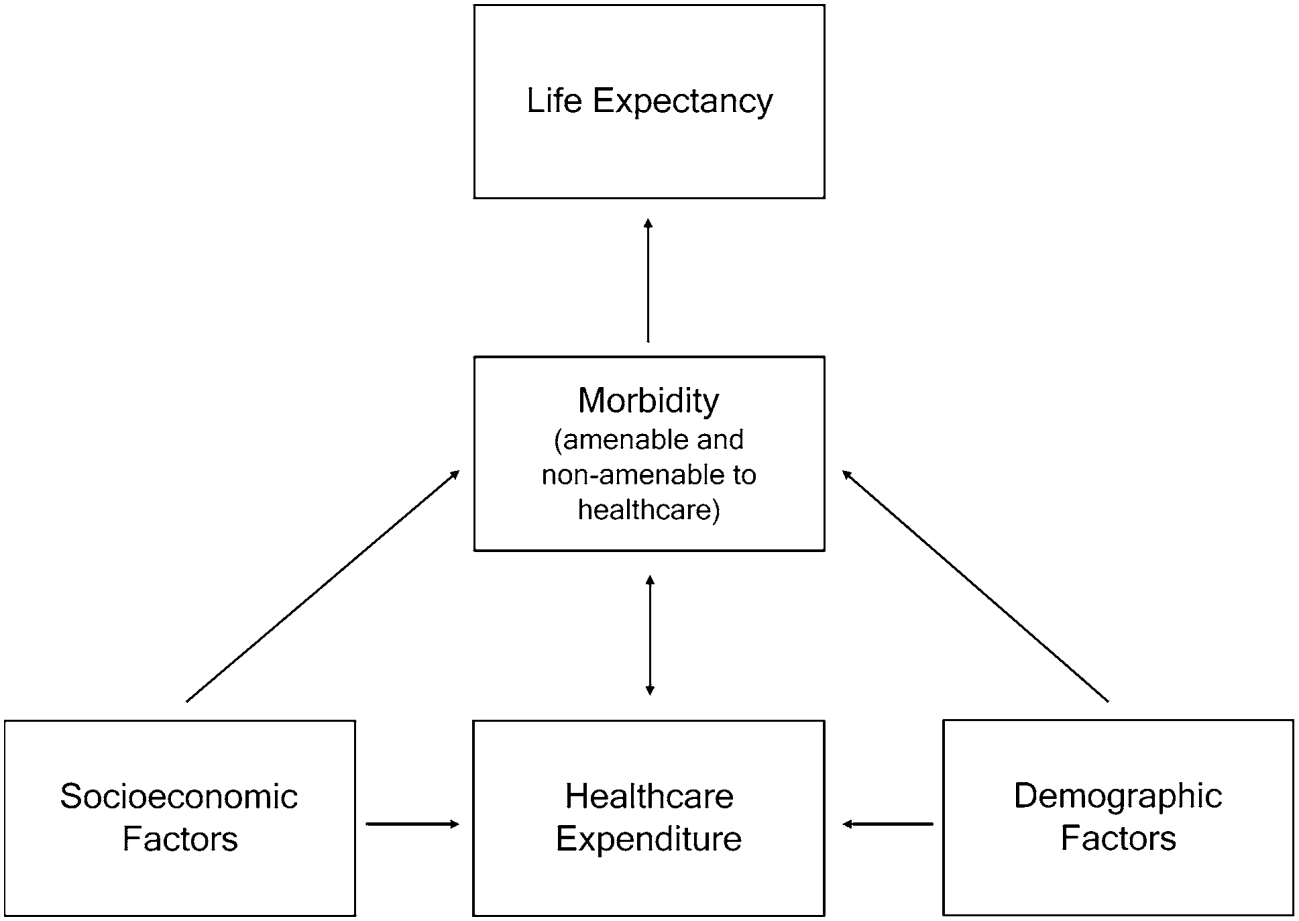
Conceptual model of the relationship between healthcare expenditure and life expectancy

Following previous work [3–5], we use two-stage least squares (2SLS) to address the issue of endogeneity. In Fig. 3, we propose a stylised conceptual model of the relationship between life expectancy and healthcare expenditure to explain the source of endogeneity and our choice of instruments.

First, we assume that life expectancy is explained by the morbidity of population. By morbidity, we intend any manifestation of poor health. Healthcare expenditure increases life expectancy by reducing morbidity or mitigating its effect thereon. We further assume that socioeconomic factors cause lifestyle-related morbidity. In addition, demographic factors affect morbidity by making it more or less costly to provide some forms of care or to achieve the same outcomes. Therefore, holding expenditure constant, an unfavourable demographic profile will lead to higher morbidity. Further, both socioeconomic and demographic factors cause expenditure because the central government redistributes funds between regions based on these factors (see, e.g. The Swedish Agency for Public Management [22, 23] for details). Socioeconomic factors will also influence healthcare expenditure through their effect on the tax base, and possibly by affecting the propensity to seek and demand care. Finally, we assume that morbidity causes higher expenditure because regions with high morbidity raise taxes or increase fees to compensate for healthcare need.

Although similar to that of previous work, this characterisation of the relationship leads us to slightly different conclusions about how it should be modelled. First, our model suggests that we should be careful when controlling for morbidity, since measures of morbidity that are affected by (amenable to) healthcare will block the path between expenditure and life expectancy.^3^ Second, some factors that on first glance appear not to directly cause life expectancy, ought perhaps to be included as demographic controls. For instance, since life expectancy is age standardised it might appear as if population age constitutes an appropriate instrument. We assume that, although age will be positively correlated with expenditure, this does not represent a ceteris paribus increase in resource use but rather a higher cost of providing care. In other words, higher age reflects an unfavourable demographic profile and belongs to the equation as a control.

We use the number of newly graduated nurses per capita as an instrument, arguing that it can be regarded as a ‘supply shift’, which affects the quantity of labour employed. In addition, the proportion of nurses aged 60–69 is used as a proxy for retirements with the same rationale. This is adjusted for the proportion of the working age population aged 60–69, to capture an over-representation of nurses close to retirement, rather than an older population overall. These instruments’ effect on expenditure can thus be seen as a change in resource use that is independent of the amount of resources required to produce a given outcome, assuming either that the price index purges the supply shift of its potential wage effect or that labour demand is elastic. The rationale for why the first instrument can be regarded as exogenous is that higher education is financed by the central government and is therefore likely to follow a national rather than a purely regional logic. That is, from the perspective of the relevant decision maker, nurses may be educated in one region to meet the labour demand of another region, but as a side effect, regions that get to educate more nurses will also have an easier time hiring. To the degree that a region might choose to train more nurses, perhaps because of need, the instrument could still be regarded as exogenous conditional on that need. In fact, considering the level of aggregation, we would expect both instruments to be correlated with other factors that influence life expectancy. This means that controls are important, not only from the perspective of improving precision, but also for ensuring that there is no residual channel through which the instruments are associated with life expectancy.

We control for socioeconomic factors, demographic factors, and for morbidity that is not amenable to healthcare. Statistics Sweden [24] show that education level is an important determinant of life expectancy and that this might be due to covariation between education, life style, and cardiovascular disease. Therefore, we consider the proportion of the population with at least 3 years secondary education as our main socioeconomic control. In addition, we include the employment rate, which is a factor in the system of regional redistribution. We attempt to measure non-amenable morbidity using the number of first time myocardial infarctions per capita and new cases of lung cancer per capita, assuming healthcare has no effect on first time incidence. We also consider the number of patients per capita hospitalised (at least once) for alcohol-related diagnoses and injuries (ICD-17), respectively. This assumes that healthcare will only affect the frequency and duration of hospitalisations per patient, not the number of patients. All socioeconomic and morbidity controls are age- and gender-standardised. As previously stated, population age is treated as a demographic control. Since mean age or the proportion of elderly in the population is clearly endogenous, however, we instead consider the annual change in mean age from inter-regional migration.^4^ The same is done for the male proportion of the population. Population density is also included as a demographic variable to reflect the increased cost of providing healthcare in rural areas. All three variables are consistent with factors used in the regional redistribution system [22]. Since regional differences in life expectancy persist within socioeconomic groups, which is particularly evident for the Norrland regions [24], we include a dummy for these regions (the five northernmost regions in Fig. 2). We also add a dummy for regions with a teaching hospital, since it is likely to be associated with our instruments, but might affect life expectancy in other ways. All data are collected from Statistics Sweden, except for the old nurses instrument and the morbidity measures, which are taken from the National Board of Health and Welfare.

**Fig. 4:**
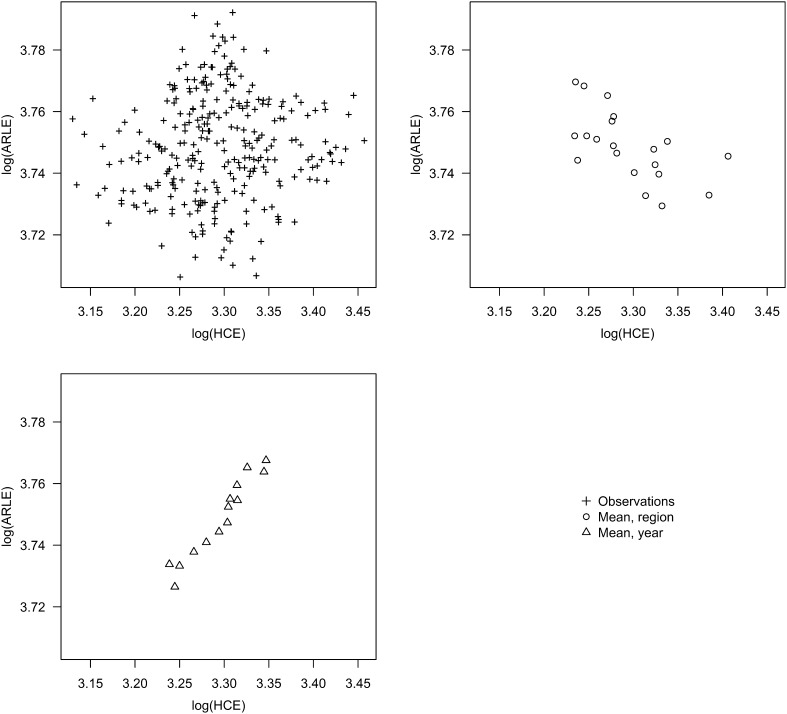
Temporal and cross-sectional relationship between healthcare expenditure and life expectancy

Regarding estimation, there are two different relationships between healthcare expenditure and life expectancy apparent in the data. Figure 4 shows that the cross-sectional relationship is negative, while the relationship over time is positive. Since the time series analysis deals with the temporal relationship, we attempt, here, to estimate the cross-sectional relationship by introducing fixed effects for periods. Region fixed effects are excluded since, without a lag structure, the long-run relationship of interest is expected to be present in the cross-sectional variation of expenditure and life expectancy, rather than the annual variation within each region. The options of a dynamic panel or an average cross-section are not considered because of the sample size. More precisely, we estimate:

3 $$\ln {\text{HC}}{{\text{E}}_{i,t}}={{\varvec{z}}^{\prime}_{i,t}} \varvec{\delta} +{{\varvec{x}}^{\prime}_{i,t}}\varvec{\theta}+{e_{i,t}},$$

4 $$\ln {\text{ARL}}{{\text{E}}_{i,t}}=\beta {\widehat {{\ln {\text{HCE}}}}_{i,t}}+{{\varvec{x}}^{\prime}_{i,t}}\varvec{\gamma}+{\varepsilon _{i,t}},$$

by least squares, where ${{\varvec{z}}_{i,t}}$ is a vector of instruments and ${{\varvec{x}}_{i,t}}$ is a vector of controls, including constants. Equation 3 is referred to as the first stage, where we examine the relevance of the instruments by imposing the restriction $\varvec{\delta}=0$. The resulting F-statistic is not compared to the standard F-distribution for 2SLS inference. Rather, a statistic exceeding 10 is commonly regarded as sufficient for rejecting that the instruments are weak, which is a reasonable rule-of-thumb with a single endogenous regressor and one or two instruments [25, 26]. For Eq. 4, referred to as the second stage, the validity of the instruments is tested using the Sargan test of over-identifying restrictions. The Wu–Hausman test is used to determine whether the OLS estimate of $\beta$ is inconsistent when compared to 2SLS. All inference is based on heteroscedasticity, autocorrelation, and cross-sectional correlation consistent standard errors using the HC3 estimator [27, 28]. All regressions are estimated in R using the ‘plm’ and ‘AER’ packages [29–31].

### Deriving the marginal cost of a life year

The marginal effect of healthcare expenditure on average remaining life expectancy can easily be translated to the marginal cost per life year in a fashion similar to that of Lichtenberg [9] or Vallejo-Torres et al. [5]:

5 $${\text{Cost}}\;{\text{per}}\;{\text{LY}}={\text{ARLE}}\frac{1}{{\frac{{\partial {\text{ARLE}}}}{{\partial {\text{HCE}}}}}}=\frac{1}{{\frac{{\partial {\text{ARLE}}}}{{\partial {\text{HCE}}}}\;\frac{1}{{{\text{ARLE}}}}}}=\frac{{{\text{HCE}}}}{\beta }.$$

This can be understood, either as (1) the average number of years left to live times the additional expenditure during each year, divided by the gain in life years, or (2) the additional expenditure per year divided by the gain in life years allocated equally across remaining life years. The second interpretation shows that no discounting is necessary, if health and expenditure are discounted at the same rate.^5^ Calculation is carried out by simply dividing healthcare expenditure by the expenditure elasticity of life expectancy ($\beta$).

## Results

### Time series analysis

The ADF and KPSS tests suggest that both healthcare expenditure and life expectancy are integrated of order one (see Online Resource 1). We proceed by estimating a VAR(3), selected based on the Hannan–Quinn information criterion. Adding one lag, a Granger test following Toda and Yamamoto [20] rejects that life expectancy is a non-cause of expenditure (*p* = 0.004) but does not reject expenditure as a non-cause of life expectancy (*p* = 0.180). This result is robust to other lag-length specifications, which are reported in Table 1. Similar results are also obtained using healthcare expenditure at OECD constant prices (see Online Resource 1).

**Table 1:** Granger non-causality tests

Lags: ${P^ \star }+m$	ARLE ↛ HCE	HCE ↛ ARLE
1 + 1	3.827^*^	1.209
2 + 1	8.499^**^	4.064
3 + 1	13.302^***^	4.887
4 + 1	14.199^***^	5.283
5 + 1	11.141^**^	3.462

The zero restriction is imposed on the coefficients of the first ${P^ \star }$ lags; the additional $m=1$ lags are added to account for the maximum order of integration. Under the null of no causality, the test statistic is ${\chi ^2}$ with ${P^ \star }$ degrees of freedom

^***,**,*^Denotes 1/5/10% significance

This may be interpreted as evidence of longevity causing healthcare expenditure, but not the other way around. The Johansen cointegration test indicates that there is no long-run relationship.^6^ This is, possibly, due to poor small sample properties, but since the evidence from the Granger tests shows that any relationship derived using this or any other method is likely to describe the effect of life expectancy on expenditure, we conclude that a time series approach to the estimation of a marginal cost per life year is not feasible.

### Panel data analysis

The second-stage regression results are reported in Table 2, where the OLS estimate of the expenditure elasticity of life expectancy is zero (0.000, *p* = 0.993). Using instrumental variables, the estimate is instead 0.080 (*p* = 0.032). If the instruments are unconditionally exogenous, the estimate should be the same without controls. We specifically hypothesised that the presence of a teaching hospital would be associated with our instruments, but Regression 2a demonstrates that this and other variables that are not significant in Regression 1 can be excluded without any substantial effect to the estimate (0.076, *p* = 0.019). We find, however, that it is sensitive to the exclusion of the remaining variables. We also consider potential heterogeneity in the impact of expenditure by re-estimating Regression 2a using population weights. This has a very small effect on the point estimate (0.079, *p* = 0.149), suggesting that there is no important interaction with population size or other related characteristics, but reduces its precision. The full results from the weighted regression are reported in Online Resource 1.

**Table 2:** 2SLS second-stage regression results

	OLS	(1)	(2a)	(2b)	(2c)	(3)
log(Expenditure p.c.)	0.000 (0.010)	0.080^**^ (0.037)	0.076^**^ (0.032)	0.067^**^ (0.032)	0.098^*^ (0.053)	0.186^**^ (0.074)
pr(Education ≥ 3 years sec.)	0.153^***^ (0.022)	0.123^***^ (0.027)	0.114^***^ (0.024)	0.118^***^ (0.024)	0.106^***^ (0.029)	0.089^***^ (0.032)
pr(Employed)	0.229^***^ (0.023)	0.233^***^ (0.023)	0.239^***^ (0.020)	0.237^***^ (0.018)	0.246^***^ (0.026)	0.265^***^ (0.032)
log(First time MI p.c.)	− 0.012^**^ (0.005)	− 0.017^***^ (0.005)	− 0.020^***^ (0.006)	− 0.019^***^ (0.007)	− 0.022^***^ (0.008)	
log(New lung cancer p.c.)	− 0.004^*^ (0.002)	− 0.004^*^ (0.002)	− 0.004^*^ (0.002)	− 0.004^*^ (0.002)	− 0.004^*^ (0.002)	
log(Alcohol patients p.c.)	− 0.020^***^ (0.002)	− 0.028^***^ (0.004)	− 0.028^***^ (0.003)	− 0.027^***^ (0.003)	− 0.030^***^ (0.005)	
log(Injury patients p.c.)	− 0.004 (0.006)	0.004 (0.007)				
Mean age^a^	0.010 (0.017)	− 0.003 (0.019)				
pr(Male)^a^	− 2.170 (1.541)	− 2.643 (1.686)				
log(Population density)	0.001 (0.001)	0.003^*^ (0.002)	0.003^***^ (0.001)	0.003^**^ (0.001)	0.003^**^ (0.001)	0.004^**^ (0.002)
d(Norrland)	− 0.017^***^ (0.001)	− 0.020^***^ (0.002)	− 0.020^***^ (0.002)	− 0.019^***^ (0.002)	− 0.021^***^ (0.002)	− 0.024^***^ (0.003)
d(Teaching hospital)	0.000 (0.001)	− 0.001 (0.001)				
Within (period) R-sq	0.785	0.750	0.751	0.758	0.729	0.359
Within (period) adj R-sq	0.763	0.725	0.730	0.738	0.707	0.314
Overall R-sq	0.895	0.877	0.877	0.881	0.865	0.609
Overall adj R-sq	0.884	0.864	0.867	0.871	0.854	0.581
Weak instrument F-stat		17.934	21.144	33.900	9.175	13.371
Wu–Hausman F-stat		4.587^**^	6.928^***^	3.205^*^	2.941^*^	25.837^***^
Sargan Chi-sq-stat		0.617	0.422			2.154
MC/life year (2016)		348,328	367,507	418,190	283,644	149,765
MC/life year (sample mean)		337,366	355,941	405,029	274,717	145,052

^a^Annual change in the variable from inter-regional migration. ‘log()’ is the natural logarithm, ‘pr()’ is the proportion of the population, and ‘d()’ is a dummy variable. MC is the marginal cost in 2016 SEK. All models are estimated with period fixed effects (i.e. year dummies) using data for 2003–2015 (*N* = 20, *T* = 13)

^***,**,*^Denotes 1/5/10% significance. Robust standard errors within parentheses

The instruments are neither weak nor invalid according to the diagnostics tests. In Regressions 2b and 2c, we examine the effect of excluding the old nurses instrument (0.067, *p* = 0.036) and the graduated nurses instrument (0.098, *p* = 0.067), respectively. Regression 3 excludes the remaining morbidity variables which increases the effect of expenditure to 0.186 (*p* = 0.012). What is interesting about this specification is that it passes the standard diagnostics tests, but it stands to reason that this estimate cannot also be unbiased. The effect of excluding the variables suggests that the instruments can only be regarded as exogenous conditional on them. For all specifications, the consistency of the OLS estimate is rejected by the Wu–Hausman test. Regarding the other variables, all have the expected effect sign or are not significantly different from zero. We also confirm that the effect of both instruments in the first stage is in line with our rationale for them; the results from the first stage are reported in Table 3.

**Table 3:** 2SLS first-stage regression results

	(1)	(2a)	(2b)	(2c)	(3)
log(Graduated nurses p.c.)	0.031^***^ (0.007)	0.033^***^ (0.007)	0.040^***^ (0.007)		0.027^***^ (0.008)
pr(Nurses, age 60–69)^b^	− 0.059 (0.039)	− 0.099^*^ (0.053)		− 0.167^***^ (0.055)	− 0.018 (0.025)
pr(Education ≥ 3 years sec.)	0.506^***^ (0.073)	0.476^***^ (0.049)	0.417^***^ (0.037)	0.484^***^ (0.055)	0.370^***^ (0.036)
pr(Employed)	− 0.094 (0.120)	− 0.255^**^ (0.106)	− 0.247^**^ (0.109)	− 0.289^***^ (0.090)	− 0.244^***^ (0.083)
log(First time MI p.c.)	0.066^***^ (0.026)	0.068^**^ (0.032)	0.084^***^ (0.028)	0.055 (0.040)	
log(New lung cancer p.c.)	0.010 (0.009)	0.016 (0.011)	0.017 (0.010)	0.006 (0.011)	
log(Alcohol patients p.c.)	0.107^***^ (0.015)	0.114^***^ (0.015)	0.108^***^ (0.012)	0.112^***^ (0.017)	
log(Injury patients p.c.)	− 0.078^***^ (0.016)				
Mean age^a^	0.161^*^ (0.086)				
pr(Male)^a^	7.748 (7.764)				
log(Population density)	− 0.026^***^ (0.003)	− 0.026^***^ (0.002)	− 0.025^***^ (0.002)	− 0.026^***^ (0.002)	− 0.021^***^ (0.002)
d(Norrland)	0.033^***^ (0.010)	0.033^***^ (0.009)	0.031^***^ (0.008)	0.036^***^ (0.010)	0.035^***^ (0.007)
d(Teaching hospital)	0.008 (0.006)				
Within (period) R-sq	0.710	0.684	0.677	0.661	0.521
Within (period) adj R-sq	0.679	0.656	0.650	0.632	0.485
Overall R-sq	0.798	0.780	0.775	0.764	0.666
Overall adj R-sq	0.776	0.761	0.756	0.744	0.641

^a^Annual change in the variable from inter-regional migration

^b^Adjusted for the proportion of the working age (25–69) population age 60–69.‘log()’ is the natural logarithm, ‘pr()’ is the proportion of the population, and ‘d()’ is a dummy variable. All models are estimated with period fixed effects (i.e. year dummies) using data for 2003–2015 (*N* = 20, *T* = 13)

^***,**,*^Denotes 1/5/10% significance. Robust standard errors within parentheses

Using Eq. 5, the estimates of the expenditure elasticity of life expectancy are transformed into a marginal cost per life year, reported at the bottom of Table 2. The function is evaluated at the expenditure per capita for Sweden in 2016 (SEK 27,827, EUR 2939),^7^ which results in a marginal cost per life year of SEK 367,507 (95% CI 200,279–2,227,010) or EUR 38,812 (95% CI 21,151–235,192) for Regression 2a. This is the preferred specification, since it includes no unnecessary controls. To shed some light on the cost per QALY, we carry out a few back-of-the-envelope calculations. Using age- and gender-specific HRQoL weights for Sweden [32], average HRQoL in 2016 was 0.855, which would imply a marginal cost per QALY of SEK 429,833 (EUR 45,394). This is roughly in line with the effect of adjusting life years for quality of life in the study by Claxton et al. [3]. If we assume the same effect as Claxton et al. [3] from including pure quality-of-life effects, then the marginal cost per QALY would be SEK 183,539 (EUR 19,383).

## Discussion and conclusion

Our analysis finds that a long-run relationship estimated using Swedish national level time series would have to be interpreted as the causal effect of life expectancy on healthcare expenditure, and is therefore not appropriate for deriving the marginal cost of a life year. However, using instrumental variable estimation, we are able to derive a cost per life year of about SEK 370,000 (EUR 39,000) from regional variations in life expectancy and healthcare expenditure. The validity of this estimate rests crucially on the validity of the instrumental variables. We show that the instruments can only be regarded as conditionally exogenous, and we are not surprised to find that some factors such as education level or population density are associated with the instruments. However, since the exclusion of morbidity has the opposite effect of what we would expect, it raises the question whether there are other confounding factors that we do not control for. In our modelling approach, we aim at including factors that are known to be important for either life expectancy, healthcare expenditure, or both. This includes a limited number of variables representing socioeconomics, leading causes of death, and population structure. An alternative approach would be to ‘control for everything’, which would reduce the risk of any residual channel through which the instruments might have an effect, but we balance this against the risk of blocking the path between the healthcare expenditure and life expectancy. There is also the issue that correlation between the variables might not only reduce the precision of estimates, but might also make it hard to disentangle their effects [8]. We are able to show, at least, that the effect signs of all variables appear sensible, which we believe lends some credibility to the model.

We could rely on the Sargan test to argue that the instruments are valid, but as we demonstrate, it is possible to produce a markedly different estimate for which the test is not rejected either. This is not all that surprising, since the test is known to sometimes have low power or be inconsistent [33]. We are able to show that excluding either one of the instruments has a modest effect on the estimate of interest, but as a test for instrument validity, this adds little to the analysis since this is what a test of over-identifying restrictions essentially does. However, if we were to pick one of the instruments, then graduated nurses is the preferred one, since it is not a proxy for what we really would like to measure, as in the case of old nurses and retirement. It has been shown that choosing instruments based on in-sample relevance might increase the risk of incorrect inference [34], but the argument here is not that the graduated nurses instrument has a stronger first stage, but that it could be the only valid one. Though we have no strong reason to suspect that the old nurse instrument is invalid, we concede that it is not an ideal proxy since, while a greater proportion of old nurses will lead to more retirements, more retirements will cause this proportion to decrease.

The discussion so far also leads to a concern about data mining. If instruments are not unconditionally exogenous and we have no reliable test to tell us which factors are necessary to condition on, then we might run the risk of including and excluding variables until we arrive at an estimate we are expecting (or hoping for). Our choice of controls is guided by a conceptual model, but there is still an element of arbitrariness, both to the model itself and to how we measure socioeconomics, morbidity, and demographic factors. If we are correct in our suspicion that it is impossible to find a truly exogenous variable at this level of aggregation, then future research might move towards a full structural model of mortality where there is a solid theoretical foundation for the inclusion of all variables.

Regarding the use of aggregate data, the rationale for an all-cause estimate at the national level is that we are probably more interested in an average opportunity cost, rather than something, e.g. disease, gender, or age specific. Routinely reported data will also facilitate updates of the analysis and allow scrutiny from relevant stakeholders [3], which could be necessary if the estimate is to inform actual decision-making. Furthermore, using aggregate expenditure we do not run into the problem of attributing expenditure to individual patients or disease areas. However, a few reasons for a more disaggregate approach can be identified. First, we find that our data-set primarily describes regional variation that does not capture the relationship of interest, which we believe is aptly illustrated by the negative relationship in Fig. 4. Studying, e.g. disease-specific expenditure introduces an additional level of variation, since regions might make different choices on which disease areas to allocate more or less funds to. This variation could turn out to be exogenous. Second, we observe that not only population age or gender, but any regressor that measures the proportion of the population in a particular group is problematic when modelling mortality. Consider, for instance, the proportion of the population with higher education. If mortality in other education groups increases then, ceteris paribus, both aggregate mortality and the proportion of the population with higher education will increase. This does not only complicate the interpretation of results, but will cause the regressor to be endogenous in the presence of autocorrelation, since it depends on past mortality. Our guess is that this bias will be small, but it would be preferable to avoid it. It is also interesting to note that fixed effects for regions might aggravate this bias by leaving only the within variation where this effect would be present. A third reason, which we have already touched upon, is that it might be possible to find an instrument that contains an element of randomisation, although it is hard to conceive of this beyond the individual level. Finally, Claxton et al. [12] raise the issue of aggregation bias when modelling all-cause mortality. That is, if the effect of expenditure is heterogeneous across different disease groups, it could lead to a difference between the all-cause expenditure elasticity and the value implied by the different disease-specific estimates. It is clear that aggregation with respect to age poses the same problem, particularly if quality of life is to be incorporated.

Concerning the approach of estimating the relationship between expenditure and mortality, the idea is to derive the opportunity cost of healthcare resource use based on how the system is currently operating, or has been operating for the last decade or so. As such, it represents an expectation on the average marginal effect of expenditure, rather than a specific (or the best) alternative use of resources. In this respect, Chen et al. [35] make an interesting contribution by showing that many interventions with long waiting lists in Ireland have ICERs well below the official threshold of EUR 45,000. When approving new interventions, reducing waiting lists for existing interventions constitutes a very concrete alternative use of resources. This concreteness lends a certain credibility that the estimated effect of a non-specific lump sum of expenditure lacks. Therefore, it could be interesting to study the marginal effect of specific resource use on mortality. Healthcare labour (e.g. nurses and physicians) is an obvious candidate, since it constitutes both a specific option of almost unlimited spending potential and very likely candidate for displacement^8^ by new interventions.

In conclusion, this study is concerned with the positive question of what the expenditure elasticity of life expectancy is and how to estimate it, but the results carry important normative implications since knowledge of the marginal cost per life year (or QALY) in the healthcare sector provides a means to interpret the results of cost-effectiveness analysis. Clearly, we need to consider that there are uncertainties associated with our estimated cost per life year, and that we do not have any reliable data to incorporate quality-of-life effects. Still, we hope that by laying the groundwork for a Swedish measure of opportunity cost in terms of health, we can now be more explicit when incorporating the results of cost-effectiveness analysis into healthcare decision-making. If resource use falls on the healthcare sector, the results reported herein indicate the potential life years forgone when dedicating resources to a new intervention rather than other healthcare services. Thus, the important policy implication of this work is that it makes possible a move towards reporting health versus health consequences rather than ICERs in economic evaluations.

## Electronic supplementary material

Below is the link to the electronic supplementary material.

Supplementary material 1 (PDF 442 KB)

Supplementary material 2 (CSV 4 KB)

Supplementary material 3 (CSV 83 KB)

## Footnotes

1 In 2016, total revenue from patient fees amounted to SEK 6.3 billion [14], covering 2.3% of total regional healthcare expenditure.

2 The council of Gotland, which has special status as both a region and a local municipality, is excluded due to limited data availability. Because of data availability issues for other variables, the panel data analysis is restricted to the period 2003–2015 (*N* = 20, *T* = 13).

3 We do not wish argue that life expectancy (mortality) depends exclusively on morbidity, but we believe that most of the effect of other variables (including expenditure) is working through it. Any potential direct links with life expectancy are accounted for by our empirical specification, although not included in our conceptual model.

4 This variable could act as an instrument for mean age, but since this is not the causal effect of interest to the study, we use it directly as a control, so as not to confound the main results or introduce unnecessary endogenous regressors.

5 If the effect of expenditure is heterogeneous across ages, then discounting could of course turn out to be important in an analysis of age-specific mortality rates, by, e.g. tilting health gains towards the future.

6 Earlier conference presentations of this work used different measures of mortality and expenditure where cointegration was present; the Granger causality results, however, similarly indicated unidirectional causality from mortality to expenditure.

7 At the average exchange rate in 2016, EURSEK = 9.4689, from the European Central Bank.

8 New interventions would not have to displace existing personnel, but simply reduce the rate at which new staff is hired.
