# Supplementary material for: Estimating the marginal cost of a life year in Sweden’s public healthcare sector
Source: Eur J Health Econ. 2019 Feb 22;20(5):751–62. doi: 10.1007/s10198-019-01039-0 (PMC6602994; doi:10.1007/s10198-019-01039-0)
Supplement: Supplementary file 1 — Supplementary material 1 (PDF 442 KB) [file 10198_2019_1039_MOESM1_ESM.pdf]

Supplement to “Estimating the marginal cost of a life year in Sweden’s public healthcare sector”

Contents

A.1 Regional remaining life expectancy 1
A.2 Construction of a healthcare price index 2
A.2.1 Time series, 1970-2016 2
A.2.2 Panel data, 2003-2016 2
A.3 Unit root tests 4
A.4 Granger tests with expenditure at OECD constant prices 4
A.5 Population weighted 2SLS 5
A.6 Drescriptive statistics 5
References 6

A.1 Regional remaining life expectancy

At the national level, we collect data on remaining life expectancy (RLE) by age and gender for 1970-2016. At the regional level, we calculate the same measure using the number of deaths (D) by region (i), year (t), age according to birth year (a), and gender (g) for 2003-2016. First, age and gender specific mortality rates are calculated according to Equation A.1

$$\delta_{i,t,a,g} = \frac{D_{i,t,a,g}}{P_{i,t-1,a-1,g} - 0.5D_{i,t,a,g} + 0.5M_{i,t,a,g}} \tag{A.1}$$

, where M is the surplus migration to the region, and P is the population. Deaths and migration are assumed to occur at the middle of the year. At age zero, each live birth contributes half a year at risk. Mortality rates are then used to derive remaining life expectancy by age and gender

$$RLE_{i,t,a,g} = \sum_{j=a}^{120} \prod_{k=a}^{j} \exp\{-\delta_{i,t,k,g}\} \tag{A.2}$$

, where $a = 100$ includes everyone a hundred years or older; this mortality rate is applied to all ages above 100. Statistics Sweden provide five-year averages of RLE at birth by gender and region. As a robustness check, we use our method to calculate RLE at birth for the periods 2002-2006, 2007-2011, and 2012-2016. The correlation between this and the Statistic Sweden series is 0.9995. Finally, in order to produce a single aggregate measure of mortality, we derive standardised average remaining life expectancy (ARLE) according to Equation A.3

$$ARLE_{i,t} = \sum_{a,g} \frac{P_{T,a,g}}{P_T} RLE_{i,t,a,g} \tag{A.3}$$

, where P_T is the national population in 2016. In this way, we capture regional and temporal variations in mortality that are not confounded by either type of variation in the age and gender structure of the population.

A.2 Construction of a healthcare price index

A.2.1 Time series, 1970-2016

Since healthcare expenditure from the OECD is only reported at current prices or constants prices using the GDP deflator, we construct a healthcare price index in order to adjust expenditure by its sector specific price level. The regional council price index (LPI) excluding pharmaceuticals and the pharmaceutical price index are available from 1980. From 2004, the LPI including pharmaceuticals is also available. All indices are plotted in Figure A.1. We note that the LPI followed the general price level quite closely up until the 1990s. We therefore assume that the GDP deflator can be used to describe the price increase of healthcare services for 1970-1979. For 1980-2003, we construct a healthcare price index by weighting the pharmaceutical price index and the LPI by the pharmaceutical and non-pharmaceutical share of government healthcare expenditure, respectively. The OECD provides pharmaceutical expenditure as share of total healthcare expenditure for 1970-2016. Let θ_T denote this share and note that

$$\theta_T = \theta_G \phi_G + \theta_P (1 - \phi_G) \Leftrightarrow \theta_G = \frac{\theta_T - \theta_P}{\phi_G} + \theta_P \tag{A.4}$$

, where θ_G and θ_P are the pharmaceutical shares of government and private healthcare expenditure, respectively, and ϕ_G is the government share of total healthcare expenditure. θ_G, θ_P, and ϕ_G are observed for 2001-2016 in the System of Health Accounts. Since θ_P and ϕ_G remained stable at around 30% and 82%, respectively, for 2001-2010 (before the redefinition of healthcare expenditure in SHA 2011), we assume that their averages can be applied to the years 1980-2000, in order to derive the pharmaceutical share of government healthcare expenditure for 1980-2003 according to Equation A.4. Our final healthcare price index, also plotted in Figure A.1 (HealthcarePI), is constructed by linking the GDP deflator (1970-1979), the weighted LPI-pharmaceutical index (1980-2003), and the LPI including pharmaceuticals (2004-2016). Figure A.2 compares the original expenditure series to the adjusted one. The adjusted series shows that the increase in real expenditure since 1970 has been slightly lower, which is due to a sharper increase in healthcare prices compared to the GDP deflator since the 1990s.

A.2.2 Panel data, 2003-2016

The regional council price index (LPI) does not capture regional variations in price level. We adjust for this using a healthcare wage index (w_i) for 2004, which is part of one of the models in the system of regional redistribution.

$$LPI_{it}^{\star} = LPI_t (0.72 w_i + 0.28) \tag{A.5}$$

, where 0.72 is the wage share of healthcare expenditure [1]. For 2004-2016, we use the LPI including pharmaceuticals. The value for 2003 is taken from the weighted LPI-pharmaceutical index, described in previous subsection.

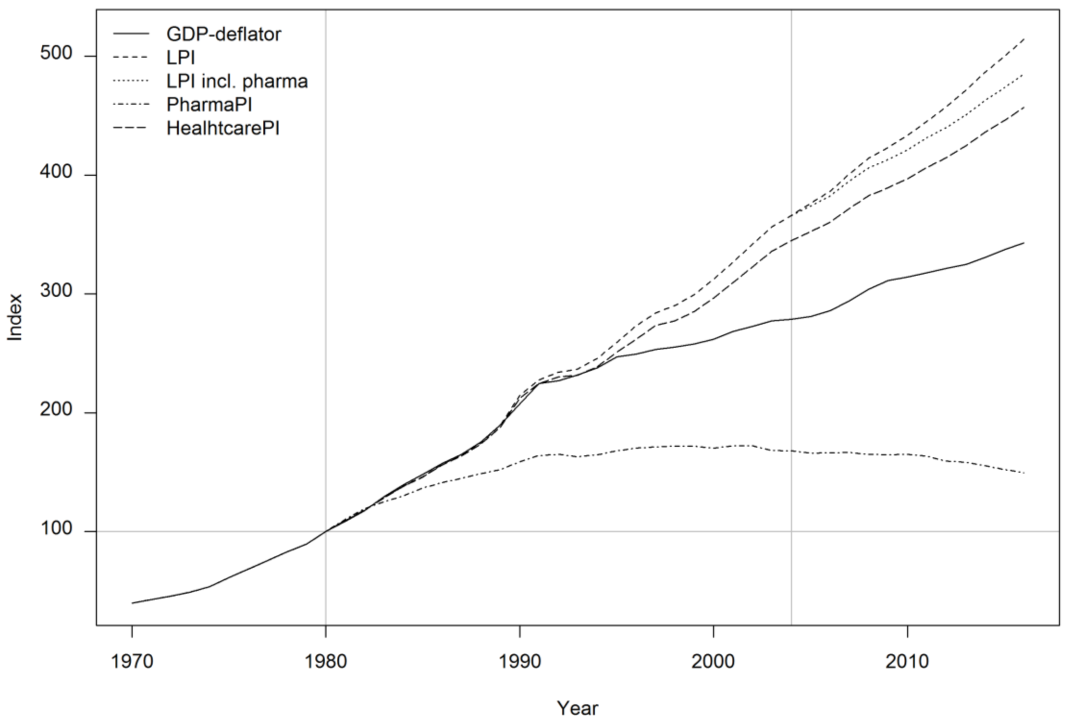

Figure A.1: Price indices

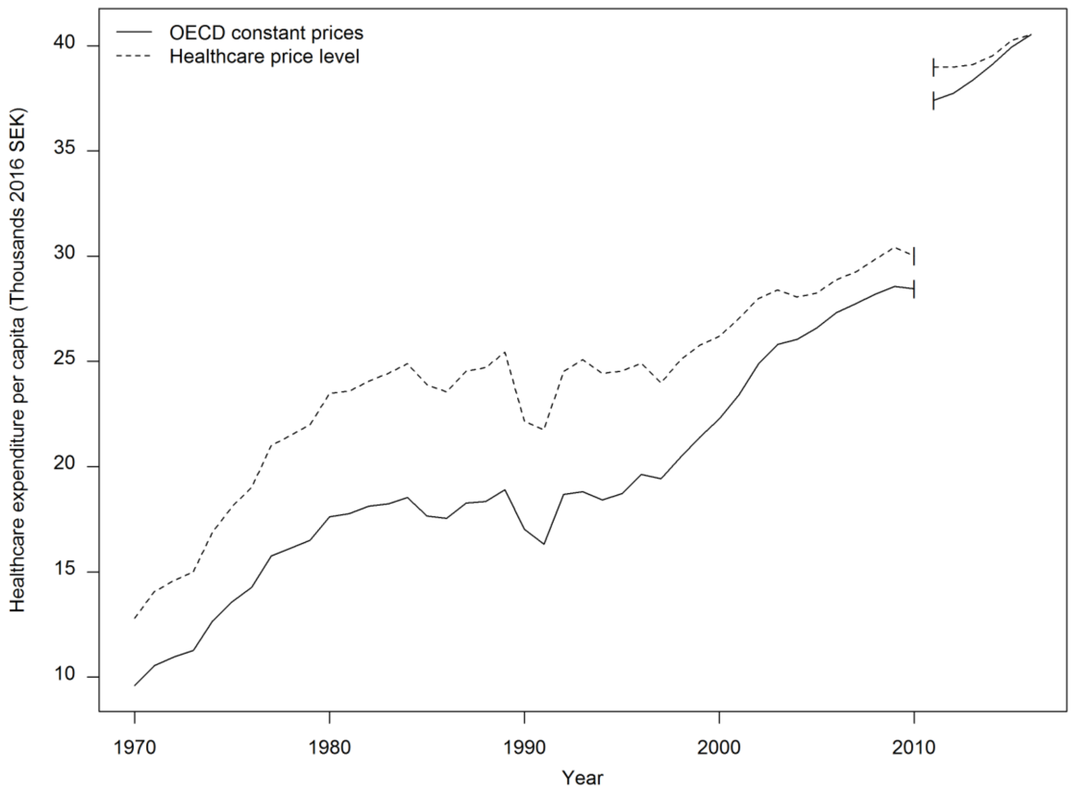

Figure A.2: Healthcare expenditure at OECD constant prices and healthcare prices

A.3 Unit root tests

We determine the order of integration of the variables for the time series analysis using the ADF-test and the KPSS-test. In the former, the null hypothesis is that the series has a unit root. In the latter, the null is that the series is stationary. The test results are reported in Table A.1.

Table A.1: Unit root tests

	ADF		KPSS	
	**constant**	**const+trend**	**constant**	**const+trend**
log(ARLE)	0.805(2)	-4.976(0)***	0.886***	0.149**
log(HCE†)	-0.925(0)	-2.019(0)	0.847***	0.122*
log(HCE)	-1.874(0)	-2.657(0)	0.801***	0.110
dlog(ARLE)	-4.237(3)***		0.224	
dlog(HCE†)	-6.731(0)***		0.165	
dlog(HCE)	-6.681(0)***		0.236	

Notes: †at OECD constant prices. '***'/'**'/'*' denotes 1/5/10% significance. For the ADF-test, the number within parentheses denotes the number of lags, selected based on the Schwarz information criterion. 'log' is the natural logarithm and 'dlog' is the first difference of the natural logarithm.

In the ADF-tests, the null cannot be rejected for the series in levels but rejected at 1% significance for the first-difference. The reverse holds true for the KPPS-tests. This strongly suggests that the series are integrated of order one, usually denoted I(1). When including a linear trend in the specification, the tests provide conflicting evidence on whether the series can be regarded as trend stationary in levels, i.e. I(0) when accounting for a linear trend. For the purpose of Granger causality testing, this does not matter; we can confidently regard the maximum order of integration as one. In the case of testing for cointegration, all variables must be I(1). However, since the specification of both a constant and linear trend in a VAR-model is implausible, we include only a constant and regard the variables as I(1).

A.4 Granger tests with expenditure at OECD constant prices

Table A.2: Granger non-causality tests, expenditure at OECD constant prices

Lags: $P^{\star} + m$	ARLE $\nrightarrow$ HCE	HCE $\nrightarrow$ ARLE
1+1	2.927*	2.191
2+1	7.950**	4.843*
3+1	12.534***	5.159
4+1	15.156***	5.875
5+1	12.032**	3.591

Notes: The zero restriction is imposed on the coefficients of the first $P^{\star}$ lags; the additional $m = 1$ lags are added to account for the maximum order of integration. Under the null of no causality, the test statistic is χ^2 with $P^{\star}$ degrees of freedom. '***'/'**'/'*' denotes 1/5/10% significance.

A.5 Population weighted 2SLS

The results from the weighted regression are reported in Table 3.A. We use the average population of each region for 2003-2016 as weights. The standard errors in the weighted regression are of the same type as in the other analyses, but use a different estimator. For this reason, the first two columns of Table 3 illustrate the effect of switching estimators (without weighting).

Table A.3: Population-weighted 2SLS second-stage regression results, Regression 2a

	(2a, HC3)	(2a, HC0)	(2a, Weighted)
log(Expenditure p.c.)	0.076**	0.076**	0.079
	(0.032)	(0.031)	(0.055)
pr(Education ≥ 3 years sec.)	0.114***	0.114***	0.081**
	(0.024)	(0.023)	(0.037)
pr(Employed)	0.239***	0.239***	0.202***
	(0.020)	(0.019)	(0.020)
log(First time MI p.c.)	-0.020***	-0.020***	-0.024***
	(0.006)	(0.006)	(0.007)
log(New lung cancer p.c.)	-0.004*	-0.004**	0.000
	(0.002)	(0.002)	(0.002)
log(Alcohol patients p.c.)	-0.028***	-0.028***	-0.030***
	(0.003)	(0.003)	(0.006)
log(Population density)	0.003***	0.003***	0.003*
	(0.001)	(0.001)	(0.002)
d(Norrland)	-0.020***	-0.020***	-0.019***
	(0.002)	(0.002)	(0.003)
Weak instrument F-stat	21.144	23.528	9.369
Wu-Hausman F-stat	6.928***	7.588***	2.878*
Sargan χ^2-stat	0.422	0.422	2.167

Notes: '***'/'**'/'*' denotes 1/5/10% significance. Robust standard errors within parentheses. [a]Annual change in the variable from inter-regional migration. 'log()' is the natural logarithm, 'pr()' is the proportion of the population, and 'd()' is a dummy variable. MC is the marginal cost in 2016 SEK. All models are estimated with period fixed effects (i.e. year dummies) using data for 2003-2015 (N = 20, T = 13).

A.6 Drescriptive statistics

Table A.4: Descriptive statistics for panel data set, years 2003 and 2015

	2003				2015			
	mean	sd	min	max	mean	sd	min	max
ARLE	41.07	0.48	40.25	42.00	42.65	0.59	41.53	43.72
HCE	25,682	1,425	23,779	28,856	28,472	1,445	26,373	31,233
Graduated nurses p.c.k.	0.43	0.13	0.24	0.63	0.47	0.12	0.27	0.79
pr(Nurses age 60-69) [b]	0.44	0.06	0.36	0.60	0.72	0.06	0.61	0.90
pr(Education ≥ 3 years sec.)	0.45	0.03	0.41	0.55	0.57	0.03	0.53	0.64
pr(Employed)	0.75	0.02	0.71	0.80	0.78	0.02	0.73	0.82
First time MI p.c.k.	5.96	0.43	5.12	6.76	3.83	0.32	3.09	4.63
New lung cancer p.c.k.	0.37	0.07	0.27	0.49	0.41	0.07	0.26	0.54
Alcohol patients p.c.k.	1.60	0.36	1.01	2.45	1.52	0.27	1.18	2.22
Injury patients p.c.k.	13.19	1.49	9.02	15.60	12.45	1.40	10.10	15.16
Mean age [a]	0.0111	0.0292	-0.0294	0.0648	0.0223	0.0448	-0.0973	0.1026
pr(Male) [a]	0.0000	0.0002	-0.0003	0.0004	0.0000	0.0003	-0.0005	0.0006
Population per km^2	44.41	61.84	2.60	285.40	49.97	74.22	2.60	342.00
d(Norrland)	5/20				5/20			
d(Teaching hospital)	7/20				7/20			
Population	445,041	475,001	127,800	1,855,726	487,061	555,239	127,053	2,214,627

Notes: [a]Annual change in the variable from inter-regional migration. [b] Divided by the proportion of the working age (25-69) population age 60-69. 'pr()' is the proportion of the population and 'd()' is a dummy variable. ARLE is average remaining life expectancy. HCE is per capita healthcare expenditure in 2016 SEK. 'p.c.k.' denotes per 1,000 persons.

References

1. The Swedish Agency for Public Management: Det kommunala utjämningssystemet - en beskrivning av systemet från 2014. (2014)
